# Comparison between random and convenience samples in a multicenter survey to evaluate medical students’ quality of life

**DOI:** 10.1371/journal.pone.0332850

**Published:** 2025-10-07

**Authors:** Paulo Sergio Panse Silveira, Patricia Zen Tempski, Fernanda Brenneisen Mayer, Sylvia Claassen Enns, Munique Peleias, Milton de Arruda Martins, Jose Oliveira Siqueira

**Affiliations:** 1 Departamento de Patologia, Faculdade de Medicina, Universidade de Sao Paulo, Sao Paulo, Brazil; 2 Centro de Desenvolvimento de Educacao Medica, Faculdade de Medicina, Universidade de Sao Paulo, Sao Paulo, Brazil; 3 Faculdade de Medicina, Pontificia Universidade Catolica do Parana, Parana, Brazil; 4 Departamento de Clinica Medica, Faculdade de Medicina, Universidade de Sao Paulo, Sao Paulo, Brazil; King Abdulaziz University Faculty of Medicine, SAUDI ARABIA

## Abstract

Evaluating medical students’ mental and physical health is challenged by difficulties in obtaining randomized samples and the limitations of convenience samples. We conducted a multicenter study to assess the educational environment, quality of life, and emotional competence of medical students. Between 2011 and 2012, a total of 1,350 randomly selected students from 22 schools and 1,201 volunteer students from 50 schools across Brazil completed all questionnaires (WHOQOL-BREF, VERAS-Q, IRI, RS-14, BDI, PSQI, ESS, IDATE, MBI, and DREEM). Monitoring, support for local researchers, and personalized feedback strategies were applied to ensure the participation of randomized students, achieving a response rate of 81.8%. The platform was also available to volunteers. The statistical analysis examined the effect of these two recruitment strategies using general linear models controlling for sex, age, body mass, course year, physical activity and metabolic equivalents, school type, city population, and location. A significance level of 5% and effect sizes estimated by Cohen’s eta-squared were applied to the variables of interest, both using the Bonferroni correction. The volunteer group had more women, fewer students from the final course years, and a larger number of students from private schools and larger cities. These variables largely explained the statistically significant differences and effect sizes observed between randomized and volunteer groups. In conclusion, although some valuable lessons and motivational strategies were identified, the considerable effort required to achieve high adherence through active outreach may not be justified, as results between random and volunteer samples showed minimal differences in questionnaire responses. Our findings suggest that future studies should consider lighter motivational strategies to reduce the burden on research teams. Data and R scripts to replicate the statistical analysis are available in Harvard Dataverse at https://doi.org/10.7910/DVN/YECV8E.

## Practice points

- This is a rare opportunity to compare the answers of a large sample of randomized and volunteer medical school students.
- To achieve a high response rate, a research team must devote effort to developing and applying motivational strategies.
- The statistical differences in the responses obtained from these two groups of students are related to the composition of the group (with volunteers including more women, fewer seniors, and more students from private schools and larger cities), but do not show significant changes in the measurements from the questionnaires.
- This absence of differences in the questionnaires suggests that the effort for randomization may not be worth it. Perhaps what is captured by questionnaires related to the quality of life or educational environment is diluted by large samples.
- In conclusion, studies using convenience samples can still obtain robust results, especially if larger samples are obtained.

## Introduction

Survey researchers often face many challenges to recruit respondents and to ensure data quality. However, the processes for developing surveys are not very often discussed [1–3]. Part of the controversy is related to random versus convenience samples. Defenders of random samples are always concerned with better coverage and unbiased data, while other researchers believe that convenience samples may better reflect local school reality [1,4–6].

While technology facilitates data collection, it is not a panacea. Electronic resources allow the involvement of a greater number of research centers and instruments [3,4,7–12], while other apply printed questionnaires to a few research centers and instruments [13–16]. A noteworthy exception is the series of studies of Roh et al. [17], on depression and academic performance, which utilized only printed copies involving 21 centers and 7,357 students, achieving over 52% response rate with the participation of 36 out of 41 existing medical schools in South Korea.

The medical course is long and demands full-time dedication, with frequent exposure to patient morbidity and mortality, which always raises in researchers the suspicion that, when using a convenience sample, those students experiencing greater levels of distress predominate among the volunteers responding to surveys, thus distorting the conclusions of studies. Applying random sampling adds complexity to the already challenging task of recruiting and motivating respondents, so this study aims to assess whether such additional effort is worthwhile.

This task must, therefore, be delegated to a network of local researchers which should, in turn, be committed to efficient data collection. However, the larger the study, the more complicated its implementation may be. In order to simplify operations and improve the adherence and compliance of local collaborators, most study designs choose a more straightforward convenience sampling, without randomization [11,18,19].

Here we evaluate Brazilian medical students’ quality of life, emotional competencies, and educational environment of random-selected students from 22 schools and volunteer students from 51 schools.

This study explores the rare opportunity to use an electronic platform recruiting a large number of students who were randomized at the same time that the project was publicized to medical schools nationwide, yielding a convenience sample comparable in size to the randomized group. For this reason, this study aimed to verify whether the assessment of psychophysical health is affected by conditions related to the recruitment method.

## Literature review

To place the present work in context, we provide a brief review of the literature with emphasis on studies that describe the situation of medical education in Brazil, as well as evaluate quality of life, well-being, or related psychological and social metrics applied to undergraduate medical students as primary participants, including the sampling methods used, sample size, and the types of questionnaire administered.

Concerns about the quality of life of medical students in Brazil have existed for some time, particularly because there has been a pronounced expansion in the number of medical schools in the country over the last two decades.

For perspective, between 2010 and 2021 the number of schools changed from 183 to 366, with 54,870 vacancies available by the end of 2021 [20]. Due to the rapid expansion, some numerical divergence is observed depending on the time of year when the count is made; Tempski et al. reported a total of 201 active medical schools with an enrollment of 110,000 undergraduate students [21], of which 48 were newly established and still had no graduating class. The remaining 153 schools, with a combined enrollment of 86,000 students, were noted by Nassif et al. [22]. During this year in which the data collection period for the present study was performed, Brazil held the second position globally in the number of medical schools, trailing behind India, which had 346 schools [23].

This expansion continues, although with conflicting numbers. Official data should be published by INEP’s Higher Education Census and the federal e-MEC system; it is announced that data collection was concluded but its results have not yet been released, and the last reports seem to be related to 2022 [24]. Estimates from other sources for 2024/2025 are around 416 schools (373 active with 40,234 admission vacancies, and another 43 awaiting authorization to begin their activities) [22] or 448 schools with 44,491 admission vacancies per year [25]. Whatever the exact numbers, Brazil surpassed India (392 schools) and China (158 schools) [22], countries that have populations six times larger than ours, and the United States, which has 184 medical schools.

Approximately 87% of the new vacancies created between 2013 and 2022 were in private institutions [26], with tuition fees ranging from R$ 5,000 to R$ 16,000 [22] (for perspective, the Brazilian minimum wage in 2025 was increased to R$ 1,518). Due to this predominantly private expansion, federal programs were created for students from rural areas and lower-income backgrounds, thereby altering sociodemographic profiles in medical training; ProUni provides full or partial scholarships that do not require repayment, while FIES is a government student loan program that must be paid back under subsidized conditions.

Considering that the medical course in Brazil lasts six years, we are dealing with more than 260,000 students facing structural weaknesses in medical education that are not only relevant to academic quality and professional preparedness, but also have direct implications for the well-being and quality of life of students, who face disparities, high financial burden, uneven learning environments, and variable support across institutions [27]. Many institutions face precarious infrastructure, overloaded preceptors, and enduring regional inequalities [26]. These changes reinforce the need for public policies that aim to regulate and monitor educational quality, infrastructure, faculty development, socioeconomic inclusion, and quality of life.

In Brazil, a system of accreditation of medical schools was established in Brazil in 2015 by the Brazilian Federal Council of Medicine (Conselho Federal de Medicina, CFM) in collaboration with the Brazilian Association of Medical Education (ABEM). The evaluation includes 80 domains with several items in educational management, programs, faculty, students, and educational resources, classifying each item as sufficient or insufficient, in 76 medical schools. Although the study considered about 71.7% of these domains sufficient, it pointed out weaknesses in mentoring programs (26.3% sufficient), student permanence support (45.3%), health care (55.3%), and quality of life programs (43.4%) [28]. In addition to the fact that this initiative has reached only a small fraction of schools in operation, the accreditation process is voluntary. Therefore, it represents a convenience sample and, since schools are evaluated only if they request it, the overall scenario is probably more deficient than what is observed in this report.

Several publications from our research group approached different aspects of quality of life, emotional well-being, and academic performance. The present article compares the metrics of a sample obtained by convenience, which was conducted in parallel and had not yet been explored before, with another of a randomized sample. From this randomized sample of Brazilian medical students we can summarize the main findings. Paro et al. [29] applied the Davis’s Multidimensional Scale of Interpersonal Reactivity (IRI), the World Heath Organization Quality of Life Questionnaire (short form, WHOQOL-BREF), and the Maslach Burnout Inventory (MBI), finding that female students reported greater empathic concern but also greater personal distress, in addition to higher burnout scores. Emotional exhaustion and depersonalization were associated with lower empathy, whereas higher personal accomplishment correlated with greater cognitive empathy. Tempski et al. [30] used a Resilience Scale (RS-14), WHOQOL-BREF, the Dundee Ready Education Environment Measure (DREEM), Beck Depression Inventory (BDI), and the State-Trait Anxiety Inventory (STAI), and observed that students with low resilience reported poorer quality of life in all WHOQOL domains, more negative perceptions of the educational environment (DREEM), and more symptoms of anxiety and depression, showing a clear dose–response relationship. Enns et al. [31] evaluated global quality of life, WHOQOL-BREF, and DREEM, concluding that the perception of the educational environment was a key determinant of quality of life and mediated its association with depressive and anxious symptoms. Mayer et al. [32] applied the BDI and STAI, showing high prevalence of depression and anxiety, especially among women and mid-course students, highlighting the need for psychological support during training. Finally, Perotta et al. [33] used the Pittsburgh Sleep Quality Index (PSQI) and the Epworth Sleepiness Scale, and found that poor sleep quality and excessive daytime sleepiness were frequent and associated with worse quality of life and increased risk of burnout. Taken together, these studies highlight the multifactorial nature of medical students’ quality of life, involving emotional, cognitive, social, and environmental dimensions, and converge in pointing to the importance of institutional attention to students’ mental health, educational environment, and living conditions.

A cross-sectional survey of 129 Brazilian medical students found very high levels of psychological distress and burnout. Women scored higher on burnout, while men more often reported previous mental disorders. Anxiety was more frequent during the course, depression predominated before entering medical school. Alcohol misuse, cannabis and ecstasy use were more prevalent in comparison with other courses, thus suggesting that medical students represent a particularly vulnerable group in Brazil [34]. A more recent Brazilian survey used a convenience sample of 10,844 undergraduate students of several fields, including 3,659 medical students that showed sharper declines in quality of life, particularly in the physical and psychological domains, compared with peers from other fields. Anxiety and depression were highly prevalent, with a substantial proportion of untreated cases, and medical students reported greater use of antidepressants and cognitive enhancers. Although they expressed higher academic satisfaction, they also faced more time-management difficulties. The group presented a more advantaged socioeconomic profile because the sample was obtained from 32 private institutions, but revealed marked racial underrepresentation, showing that structural inequalities in access to medical education still remains [35]. Only one longitudinal study with a two-year follow-up was identified, in which 312 respondents completed questionnaires every semester approaching quality of life (WHOQOL-BREF), mental health (DASS-21), and religiousness (DUREL) instruments. As in other studies, medical students showed high levels of emotional disorders with depression, anxiety, stress, low income, female gender, early stages of medical training, and non-white ethnicity associated with poorer mental health and quality of life at follow-up [36].

Recent evidence underscores that these concerns remain unresolved not only in Brazil. For instance, Galgam et al. (2024) applied the WHOQOL-BREF among health students (n=349) and reported that dentistry students tend to achieve higher QoL scores compared with pharmacy and nursing students, with younger students in early years showing lower scores [37]. Putri et al. (2025) found a decline in QoL among nursing students (n=147), particularly in selected domains, using descriptive statistics and Pearson correlation [38]. Among health professionals in Gaza (n=1850), QoL was considered moderate despite the use of validated scales [39]. Other investigations reported strong associations between burnout, traumatic stress, and the practice environments of nursing students (n=341) [40]. In Austria, Huber et al. (2024) evaluated medical students before and after the COVID-19 pandemic, finding an increase in depressive and anxious symptoms, deterioration in subjective well-being, and perception of heavier workload and stress, with course year emerging as a predictor of QoL, particularly lower scores among those in early years [41].

Meta-analyses reveal similar trends. Pacheco et al. synthesized 59 studies with over 18,000 students and reported high prevalences of depression (30.6%), common mental disorders (31.5%), burnout (13.1%), problematic alcohol use (32.9%), stress (49.9%), poor sleep quality (51.5%), and anxiety (32.9%), with academic overload and lack of support as major correlates [42]. Soares et al. analyzed 14 studies and found a pooled prevalence of 43.3% for common mental disorders, associated with dissatisfaction with the course, desire to drop out, and sleep problems [43]. Solis and Lotufo-Neto identified predictors of quality of life in Brazilian medical students, showing lower scores among women and a decline across the years of training; negative predictors included depression, burnout, sleep difficulties, and chronic illness, while resilience, empathy, and physical activity were protective factors [44]. A recent meta-analysis identifying five eligible studies including 1,819 participants. The analysis compared students in the pre-clinical and clerkship cycles using the WHOQOL-BREF and found that medical training was associated with a decline in quality of life, particularly in the psychological and social domains. These findings indicate that the most critical impact of medical education on students’ well-being is concentrated in mental health and social aspects, suggesting the need for institutional measures to mitigate these effects [45].

Internationally, a meta-analysis by Erschens et al. including studies from multiple countries confirmed the heavy burden of burnout among medical students, with prevalence rates ranging from 7% to 75% depending on the context and measurement instruments [46], which suggests that this sort of problems is not a Brazilian uniqueness. A review mapped the literature on mental health among medical students in South Africa and identified eight eligible studies published between 2010 and 2023 [47]. Despite the limited evidence, the review also showed high rates of anxiety, depression, and burnout, with risk factors including academic overload, financial stress, cultural and linguistic isolation, and exposure to social inequalities and campus unrest.

In summary, many studies rely on non-probabilistic, convenience-based sampling (often via online or social media recruitment) without a systematic comparison of random versus volunteer respondents [35]. In Brazil, although numerous investigations have been conducted, most rely on convenience samples, often online, which limits representativeness. Cross-sectional designs with nonprobabilistic recruitment predominate, and there is substantial heterogeneity in instruments and limited standardization, hampering direct comparisons across studies. Systematic reviews and meta-analyses confirm these patterns and emphasize gender and year-of-course as key predictors of Quality of Life (QoL). However, few studies directly examine how recruitment strategy itself (randomized versus volunteer participation) may affect findings, leaving a gap regarding the validity and generalizability of survey-based evidence. The present study addresses this gap by analyzing large, parallel samples of randomized and volunteer medical students in a nationwide Brazilian survey.

## Methods

In Portuguese, the acronym VERAS stands for “Vida do Estudante e Residente na Área da Saúde" (Life of the Student and Resident in the Health Area). This project began in 2010 with support of the Brazilian Ministry of Education, aiming at a cross-sectional, nationwide, multicenter study involving only medical undergraduate students, carried out by a core coordinating team and local researchers at each collaborating center.

The present study was classified as low-risk, and the research protocol number 181/11 was approved by the Research Ethics Committee of the School of Medicine, University of São Paulo (Comitê de Ética em Pesquisa da Faculdade de Medicina da Universidade de São Paulo). All participating medical schools were notified of this approval to ensure adherence to the study, including: Universidade Federal do Rio de Janeiro, Universidade Federal de Ciências da Saúde de Porto Alegre, Universidade Estadual do Piauí, Faculdade de Medicina de Petrópolis, Faculdade de Ciências Médicas da Paraíba, Pontifícia Universidade Católica de São Paulo, Universidade Federal do Ceará, Universidade Federal de Goiás, Universidade Federal de Mato Grosso do Sul, Escola Baiana de Medicina e Saúde Pública, Faculdade de Medicina de Marília, Faculdade de Medicina de São José do Rio Preto, Faculdade Evangélica do Paraná, Faculdade de Medicina do ABC, Fundação Universidade Federal de Rondônia, Pontifícia Universidade Católica do Rio Grande do Sul, Universidade Federal do Tocantins, Universidade Federal de Uberlândia, Universidade Estadual Paulista Júlio de Mesquita Filho, Centro Universitário Serra dos Órgãos, Universidade de Fortaleza, and Universidade de Passo Fundo.

### Sample size and setting

The sample size of 1,152 students (576 men and 576 women) was computed to an effect size of 0.165 on differences between male and female participants, and statistical power of 80% at 5% significance level [48].

At the time of planning data collection, Brazil had a universe of 183 active medical schools with 86,000 students [22,25], since medical training in Brazil requires 6 years divided into periods of 2 years each (namely basic sciences, clinical sciences, and clerkship), local researchers of medical schools that agreed to participate were instructed to randomly select at least 60 students in clusters (5 males and 5 females per program year), for a total of 1,320 students. 1,650 students were effectively randomized, as we expected additional losses of 25%. From the outset, alongside agreements with local researchers to recruit randomized participants, we made the platform available to all students in participating medical schools, following their request that non-selected students also would have the opportunity to participate. We anticipated that announcing the electronic platform would be attractive to students interested in accessing a system that provided an automatic report with their personal scores and comparative population data upon completion.

### Data collection

A bespoke electronic platform was developed to manage questionnaires. For the volunteer group, there were no controls related to gender, training period, age or other stimulation except for public announcements of the running study in the medical schools.

Data collection was performed from August 2011 to August 2012. The registration procedure was identical for both volunteer and randomized samples. An online informed consent was presented prior to respondent participation in the study. Each respondent was required to answer some questions related to physical activity, personal data, and some validated questionnaires.

To capture a broad construct such as psychophysical health, we used several questionnaires commonly employed in the literature. The 14 questionnaires totaled 285 questions, and were presented in random order to different respondents as to minimize influences caused by the sequence of questions as well as respondent exhaustion. The investigated aspects can be grouped into:

- Socio-demographic Data: Comprising a socio-demographic questionnaire that investigates respondent characteristics such as age, gender, current course year, city of origin, name of the medical school enrolled, and entry system into the course;
- Quality of Life: A group of three instruments, including a self-assessment of Quality of Life (a subjective rating from 0 to 10 for overall quality of life and quality of life in the medicine course), the World Heath Organization Quality of Life Questionnaire (short form, WHOQOL-BREF), and Student Quality of Life Questionnaire (VERAS-Q, our customized questionnaire), assessing the student’s perception of their overall quality of life and life within the medical course;
- Empathy: Evaluated through Davis’s Multidimensional Scale of Interpersonal Reactivity (IRI);
- Resilience: Analyzed using the Wagnild and Young’s Resilience Scale (RS14), abbreviated scale;
- Sleep: Comprising a self-assessment of sleep, the Pittsburgh Sleep Quality Index (PSQI), and the Epworth Sleepiness Scale, analyzing aspects of sleep such as quality and quantity of sleep and daytime sleepiness;
- Depressive and Anxiety Symptoms: Investigated through two questionnaires, Beck Depression Inventory (BDI) and the State-Trait Anxiety Inventory (STAI);
- Learning Environment: Assessed by the Dundee Ready Education Environment Measure (DREEM), providing a global evaluation of the learning environment;
- Burnout: Analyzed by the Maslach Burnout Inventory (MBI);
- Physical Activity: Assessed through a qualitative questionnaire regarding the type of physical activity and the number of hours dedicated to sports activities, from which Metabolic Equivalents (METs) were computed for the present analysis.

Students had a 10-day deadline to complete the survey. We required this deadline for completion because many questions concerned events which had occurred in the previous two weeks. However, while in the 10-day period, it was possible for the respondent to interrupt and resume the survey at any time. Questionnaires were presented in random order, which differed across participants; once the order was established, each questionnaire could only be started after completing the previous one.

Due to the platform design, few instances of missing data occur because respondents were always alerted before each questionnaire was accepted and recorded. In addition, a friendly reminder of the deadline for completion was sent via email every two days while there were incomplete questionnaires. However, any respondent could abandon the questionnaires and, after 10 days, no more reminders were sent to them.

Between questionnaires, a humorous cartoon related to medical education and practice authored by two Brazilian cartoonists (Arionauro da Silva Santos and Dr. Ronaldo Cunha) was shown at to hold participants’ interest. After the completion of the whole questionnaire set, students received feedback about their personal answers.

### Statistics and other analyses

From data obtained from students from two recruitment methods (randomized and volunteers), personal data, city location of the school, and the questionnaire scores were selected for analysis. The objective of this study is attitudinal, i.e., to determine whether the responses to the employed measurement instruments differ on average when a student is recruited randomly versus when they voluntarily choose to participate in the study. For this, the recruitment method (the dependent variable, randomized or convenience samples) can be modeled as a function of the questionnaire responses (interest variables) and variables related to the students (controls). The control variables were biological determinants: sex and age; well-being context: BMI, physical activity, and METs estimate; school environment: school type and course year; and city environment: population and location.

Main effects general linear models (GLM) with only the questionnaire scores or including the controls were analyzed. Given the large sample size (more than 1000 respondents), concerns about the normality assumption were minimized, as general linear models are robust to such violations under the Central Limit Theorem [49]. The initial model included only questionnaire global scores and, then, the questionnaires that are structured in subdomains were also assessed. After that, a *post hoc* analysis of randomized and volunteer can be performed regarding the main associations found by GLM to show in which aspects these groups may differ.

Significance level of 5% and effect sizes of Cohen’s partial eta-squared ($\eta^{2}$) with Bonferroni correction were applied to the independent variables of interest. These effect sizes represent the percentage of outcome variance explained by each independent variable, whether covariates or factors, used as variables of interest or control. To evaluate the effect size, we calculated its confidence interval 95%, adopting a tolerance of 0.01% (effect sizes rounded to two decimal places). Effect sizes below this threshold of 0.01% were considered negligible, rounded to null value in the interval and are therefore regarded as nonexistent.

Qualitatively, we also applied the SWOT analysis ( **S**trengths, **W**eaknesses, **O**pportunities, and **T**hreats) for strategic planning [50]. The objective was to match the strengths to opportunities and to convert weaknesses or threats into strengths or opportunities. We requested that all researchers participate in the SWOT at the end of the data collection period.

## Results

### Participant recruitment and sample composition

A total of 1,650 random students were selected. They were from 31 schools initially committed; 9 schools were excluded (4 could not initiate data collection due to lack of institutional support, and 5 would not reach a minimum response rate of 50%), thus remaining 22 schools from which 1,350 random-selected students completed all questionnaires and were enrolled in this study.

There were at least 2 schools from each of the 5 geographic Brazilian regions; 13 are public schools and 9 are private schools; half of the schools are from noncapital areas and half from large metropolitan areas. This is a mixed group of schools, whether applying traditional, PBL, or blended methods. The number of years in operation for each school also varied: 6 are new schools founded during the past 15 years, 14 were founded between 43 and 63 years ago, and 2 others are the most traditional medical institutions in Brazil founded over a century ago.

In addition to the randomized sample, many non-selected students also chose to participate after the platform was announced to medical schools, resulting in a voluntary sample that was nearly balanced with the randomized one. Among 2,353 volunteers, 1,224 responded to all 14 questionnaires, but 23 answered that had already completed their undergraduation. Consequently, 1,201 volunteers were included in the present analysis for comparability purposes.

This criterion of completeness was applied to volunteers to ensure analytic consistency and comparability between groups because randomized students were required to complete the full set to be enrolled, while volunteer participants could freely desist at any point. In addition, among the excluded 1,129 incomplete submissions from volunteers, only 182 reached at least half of the questionnaires. It was also taken into account that partial responses would lead to inconsistently missing data and artificially lower scores because the instruments were presented in aleatory order.

These included 1,201 volunteers were students from 50 different schools, 646 from 21 schools in common with random-selected students, and 555 from 29 schools scattered across the country. Among these 29 schools, 19 are represented by only 1 to 3 volunteers, while another 9 have between 7 and 94 volunteers. One school, on the initiative of its administrator, recruited almost all of its students (263 respondents). These schools are also distributed between public and private schools and are located in non-capital or metropolitan areas.

**Fig 1:**
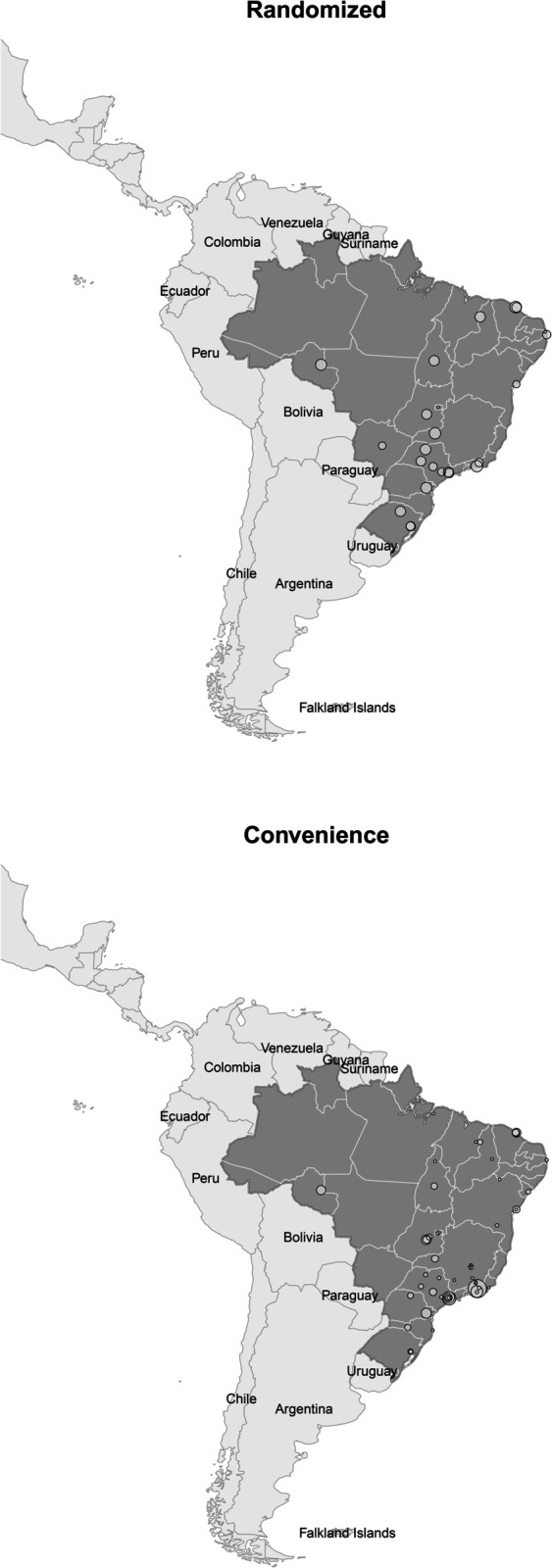
Location of Brazilian medical schools for randomized and volunteer students; circle diameter corresponds to the number of respondents. Base map: Natural Earth, 1:50m cultural vectors, public domain (https://www.naturalearthdata.com/, accessed in August 13th, 2025 [58]).

The location of the 22 schools from which randomized students and 50 schools that contributed with volunteers is shown in Fig 1. A brief descriptive comparison of randomized and volunteer students is shown in Table 1.

**Table 1:** Sample characteristics of randomized (R) and volunteer (V) students. Biological sex, Age in years, BMI: body mass index, Activity: hours per week dedicated to physical activity, METs: metabolic equivalent estimated of Kcal consumed per day, Year: undergraduation current medical course year, School: type of school, Location: capital or non-capital areas, City Pop.: population of city where the school is located.

Sex:	Female	Male	n			
**R**	714 (52.89,%)	636 (47.11,%)	1350
**V**	762 (63.45,%)	439 (36.55,%)	1201
**Age:**	**Mean**	**sd**	
**R**	22.76	3.01	
**V**	22.57	3.27	
**BMI:**	**Mean**	**sd**	
**R**	23.14	3.46	
**V**	23.03	3.71	
**Activity:**	**no.sports**	**< 1 hour**	**1 to 2**	**2 to 3**	**3 to 4**	**> 4 hours**
**R**	526 (38.96%)	75 (5.56%)	160 (11.85%)	172 (12.74%)	153 (11.33%)	264 (19.56%)
**V**	510 (42.50%)	58 (4.83%)	140 (11.67%)	144 (12.00%)	135 (11.25%)	213 (17.75%)
**METs:**	**Mean**	**sd**				
**R**	1647.96	2008.31				
**V**	1455.67	1875.29				
**Year:**	**1**	**2**	**3**	**4**	**5**	**6**
**R**	203 (15.04%)	256 (18.96%)	251 (18.59%)	240 (17.78%)	187 (13.85%)	213 (15.78%)
**V**	200 (16.65%)	300 (24.98%)	281 (23.40%)	199 (16.57%)	127 (10.57%)	94 (7.83%)
**School:**	**Private**	**Public**				
**R**	469 (34.74%)	881 (65.26%)				
**V**	748 (62.28%)	453 (37.72%)				
**Location:**	**Capital**	**non-Capital**				
**R**	878 (65.04%)	472 (34.96,%)				
**V**	665 (55.37%)	536 (44.63%)				
**City Pop.:**	**< 500 thousands**	**500 th., 1mi.**	**1mi., 5mi.**	**> 5mi.**		
**R**	363 (26.89%)	407 (30.15%)	428 (31.70%)	152 (11.26%)		
**V**	376 (31.31%)	258 (21.48%)	317 (26.39%)	250 (20.82%)		

### Response effort

For randomized students, a collaborative effort was undertaken by the core team and local researchers, which manifested in the ongoing monitoring of the questionnaires obtained throughout the study. This effort to recruit randomized students was comprehensive and employed various outreach methods, including personal invitations, emails, letters, phone calls, and messages through social media. Members of the core group dedicated time to verify and contact school administrators, encouraging them to participate in data collection with their students.

**Fig 2:**
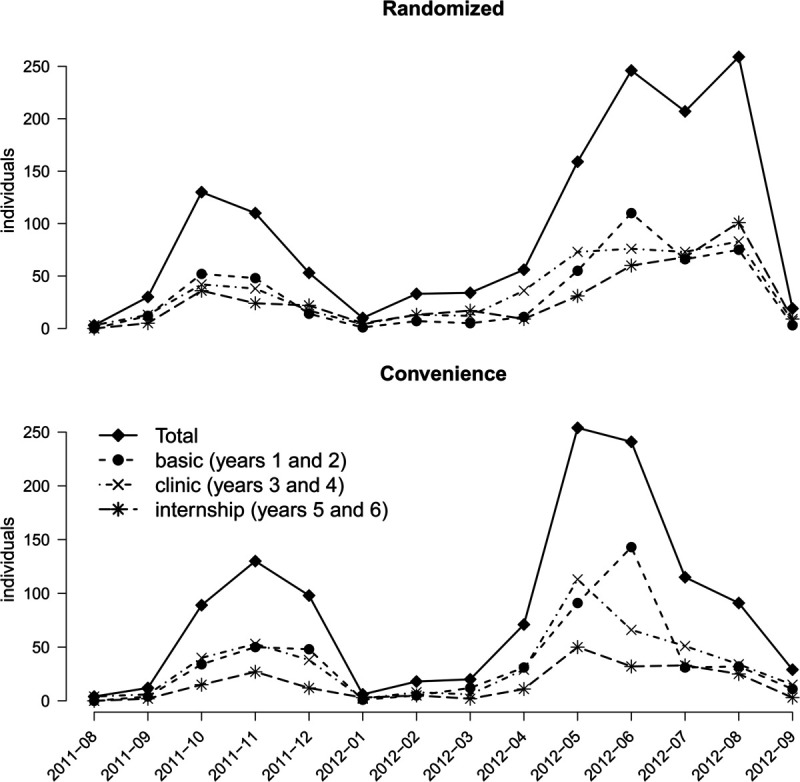
Data collection of randomized students (number of respondents along time) from the three medical course cycles (basic, 1st and 2nd; clinical, 3rd and 4th; and clerkship, 5th and 6th years). Two waves of respondents, were observed, the second coincident with the “adopt a school" strategy (see text). It was also observed that randomized respondents from the last course cycle, along the second wave, seemed to show a slower and progressive reaction to the estimuli than that obtained with the basic and clinical science cycles.

Data collection occurred between August 2011 and August 2012. Throughout this period, additional support strategies were implemented, such as regular meetings, weekly reports displaying student registrations and questionnaire follow-ups, guidance for data collection, and monthly newsletters to local researchers. Despite these measures, the pace of data collection was not consistent (Fig 2). Towards the end of 2011, as expected, there was a decline in respondents, likely influenced by approaching holidays and summer vacation in Brazil. However, this slower pace persisted into the first months of 2012. It was during this period that we introduced the “adopt a school" strategy, recognizing that local researchers were losing track of their students or feeling disconnected from the core group of researchers. To foster a continuous connection between remote researchers and the core group, this strategy involved personalized and continuous contact between each local researcher and a specific member of the core group responsible for providing tailored follow-up or solutions based on the local researcher’s needs. We attribute the observed second wave of respondents to the implementation of this strategy. Some schools, in fact, only became active after the “adopt a school" strategy was implemented.

### Questionnaire completion

Students who answered the whole set of questionnaires at once spent an average of 73 minutes to complete the task, but time distribution (not shown) was highly asymmetrical due to students who discontinued answering a questionnaire, returning to it a few days later (a few students scattered their access along the allowed 10 days). Median is the most appropriate measuring, showing that each questionnaire was typically answered in 1 to 5 minutes; the most time-consuming questionnaires were VERAS-Q and DREEM (median of 7 minutes).

Table 2 summarizes randomized and volunteer responses. In addition to the sociodemographic and physical activity questions, the questionnaires and their subdomains are as follows:

1. GQol: a subjective rating from 0 to 10 given by the respondent for their overall quality of life.
2. CQol: a subjective rating from 0 to 10 given by the respondent for their quality of life in relation to the medical course.
3. WHOQOL: the World Health Organization Quality of Life Questionnaire (short form), with the following subdomains:
  - QoL: a rating from 1 to 5 for quality of life.
  - health: a rating from 1 to 5 for satisfaction with health.
  - phys: physical health.
  - psy: psychological health.
  - soc: social relationships.
  - env: environmental health.
4. DREEM: the Dundee Ready Educational Environment Measure, with the following subdomains:
  - learn: perception of learning.
  - teacher: perception of teachers.
  - self: academic self-perception.
  - env: perception of atmosphere.
  - soc: social self-perception.
5. VERASQ: Student Quality of Life Questionnaire, developed by the local researcher group, with the following subdomains:
  - time: time management and dedication to activities other than the course.
  - psy: positive and negative feelings, concentration, support, level of demand, and self-esteem.
  - phys: health care, sleep, leisure, physical activity, and appearance.
  - env: organization of courses, relationships with peers, teachers, and the institution.
6. IRI: Davis Interpersonal Reactivity Index, with the following subdomains:
  - empathy: empathetic concern.
  - perspec: perspective-taking.
  - distress: personal distress.
7. RS14: Wagnild and Young’s Resilience Scale, with the following subdomains:
  - selfreliance: self-reliance.
  - meaningful: meaningfulness.
  - equanimity: equanimity.
  - perseverance: perseverance.
  - aloneness: existential aloneness.
8. BURNOUT: Maslach Burnout Inventory, with the following subdomains:
  - exhaut: emotional exhaustion.
  - deperson: depersonalization.
  - accomp: personal accomplishment.
9. PSQI: Pittsburgh Sleep Quality Index, with the following subdomains:
  - quality: subjective sleep quality.
  - latency: sleep latency.
  - duration: sleep duration.
  - efficiency: sleep efficiency.
  - disturbance: sleep disturbance.
  - medication: use of sleep medication.
  - dysfunction: daytime dysfunction.
10. STAI_state and STAI_trace: State-Trace Anxiety Inventory.
11. BDI: Beck Depression Inventory.
12. EPWORTH: Epworth Sleepiness Scale.

**Table 2:** Summary of randomized (R) and volunteer (V) responses to questionnaire global and subdomain scores: mean (standard deviation). See text for questionnaire descriptions.

Questionnaire	Group	Global	Subdomains			
**GQoL:**	R	7.86 (1.27)				
	V	7.65 (1.37)				
**CQoL:**	R	6.51 (1.56)				
	V	6.20 (1.65)				
**WHOQOL:**			**QoL**	**health**	**phys**	
	R	63.58 (12.84)	3.67 (0.86)	3.48 (0.99)	65.22 (14.70)	
	V	61.29 (13.67)	3.56 (0.89)	3.32 (1.00)	62.56 (15.10)	
			**psy**	**soc**	**env**	
	R		61.72 (15.69)	63.56 (19.89)	63.82 (14.08)	
	V		59.69 (16.55)	60.82 (21.38)	62.09 (14.60)	
**DREEM:**			**learn**	**teacher**	**self**	
	R	119.41 (27.12)	27.64 (7.55)	27.69 (6.91)	18.88 (5.00)	
	V	116.65 (28.01)	27.21 (7.81)	27.29 (7.24)	18.30 (5.16)	
			**env**	**soc**		
	R		29.61 (7.68)	15.59 (4.42)		
	V		28.68 (7.85)	15.15 (4.52)		
**VERASQ:**			**time**	**psy**	**phys**	**env**
	R	135.41 (22.31)	32.79 (7.85)	33.91 (7.14)	22.67 (5.18)	46.05 (7.35)
	V	131.73 (22.90)	31.68 (7.78)	32.59 (7.46)	21.86 (5.12)	45.60 (7.59)
IRI:			**empathy**	**perspec**	**distress**	
	R	69.96 (9.24)	26.08 (4.82)	24.66 (5.04)	19.22 (4.22)	
	V	70.40 (9.14)	26.20 (4.83)	24.64 (4.92)	19.55 (4.37)	
**RS14:**			**self reliance**	**meaningful**	**equanimity**	
	R	78.66 (12.40)	28.25 (4.56)	17.46 (3.07)	11.23 (2.08)	
	V	77.80 (12.22)	27.99 (4.56)	17.28 (3.06)	11.05 (2.04)	
			**perseverance**	**aloneness**		
	R		10.64 (2.59)	11.09 (2.53)		
	V		10.60 (2.54)	10.88 (2.56)		
**BURNOUT:**			**exhaust**	**deperson**	**accomp**	
	R	69.03 (13.51)	26.73 (9.80)	8.55 (5.74)	33.75 (7.61)	
	V	68.67 (13.20)	27.55 (10.01)	8.07 (5.76)	33.05 (7.58)	
**PSQI:**			**quality**	**latency**	**duration**	**efficiency**
	R	6.74 (2.94)	1.33 (0.74)	1.13 (0.93)	1.34 (0.80)	0.26 (0.66)
	V	7.26 (3.18)	1.43 (0.73)	1.21 (0.97)	1.38 (0.81)	0.31 (0.72)
			**disturbance**	**medication**	**dysfunction**	
	R		1.02 (0.57)	0.19 (0.59)	1.48 (0.82)	
	V		1.11 (0.99)	0.22 (0.66)	1.60 (0.83)	
**STAI_state:**	R	43.66 (11.65)				
	V	45.75 (12.20)				
**STAI_trace:**	R	45.49 (11.67)				
	V	47.07 (12.24)				
**BDI:**	R	9.38 (7.00)				
	V	10.78 (7.54)				
**EPWORTH:**	R	10.26 (3.90)				
	V	10.74 (4.09)				

### Effects of recruitment methods

We adopted a standardized layout across Tables 3 to 10. These tables present results of general linear models (GLM) applied to recruitment strategies (outcome: randomized or volunteer) and questionnaires (variables of interest: global scores or domains) under two conditions: without and with control variables, the latter showing which effects remain after adjustment for covariates. The lines labeled GLM correspond to the *omnibus* model test, associated with the *F* statistics, corresponding *p* value and $\eta^{2}=R^{2}$; here, rejection of the null model indicates the existence of all proposed models. In addition, for every variable of interest, we report the statistical significance and effect sizes. While statistical significance unnecessary verb informs that an effect exists (*p*-value), the effect size through the 95% confidence interval of Cohen’s eta-squared evaluates its magnitude ($\eta^{2}$, expressed in percentage); statistically significant results and non-null effect sizes are highlighted in gray.

**Table 3:** Global scores: general linear model without and with control variables; statistical significance (*p* values) and effect sizes (confidence interval of Cohen’s partial eta-squared, $\eta^{2}$ in percentage) with Bonferroni correction for 5% significance level. Statistically significant differences and the presence of effect sizes are shaded in gray. Outcome is the recruitment methods (randomized or volunteers). GLM line is the overall model test (*F*, associated with $\eta^{2}=R^{2}$).

	Without control variables				With control variables			
	F	df	p	$\eta^{2}$ (%)	F	df	p	$\eta^{2}$ (%)
**GQoL**	0.99	1	>0.9999	[0.00, 0.08]	0.76	1	>0.9999	[0.00, 0.05]
**CQoL**	4.19	1	0.5297	[0.00, 0.95]	2.02	1	>0.9999	[0.00, 0.14]
**WHOQOL**	0.02	1	>0.9999	[0.00, 0.00]	0.22	1	>0.9999	[0.00, 0.02]
**DREEM**	$1.1\cdot 10^{-04}$	1	>0.9999	[0.00, 0.00]	6.56	1	0.2302	[0.00, 1.18]
**VERASQ**	$4.7\cdot 10^{-05}$	1	>0.9999	[0.00, 0.00]	0.91	1	>0.9999	[0.00, 0.06]
**IRI**	0.86	1	>0.9999	[0.00, 0.07]	0.09	1	>0.9999	[0.00, 0.00]
**RS14**	2.33	1	>0.9999	[0.00, 0.76]	1.65	1	>0.9999	[0.00, 0.12]
**BURNOUT**	11.55	1	0.0089	[0.00, 1.53]	1.13	1	>0.9999	[0.00, 0.08]
**PSQI**	2.13	1	>0.9999	[0.00, 0.74]	0.50	1	>0.9999	[0.00, 0.04]
**STAI_state**	1.63	1	>0.9999	[0.00, 0.68]	$2.3\cdot 10^{-03}$	1	>0.9999	[0.00, 0.00]
**STAI_trace**	0.98	1	>0.9999	[0.00, 0.08]	4.81	1	0.6236	[0.00, 1.04]
**BDI**	5.43	1	0.2589	[0.00, 1.05]	2.02	1	>0.9999	[0.00, 0.14]
**EPWORTH**	3.66	1	0.7277	[0.00, 0.90]	1.12	1	>0.9999	[0.00, 0.08]
**GLM:**	**4.36**	**13, 2537**	** $1.12\cdot 10^{-06}$ **	**[1.59, 3.81]**				
				**Sex**	11.14	1	0.0188	[0.00, 1.51]
				**Age**	$5.0\cdot 10^{-03}$	1	>0.9999	[0.00, 0.00]
				**BMI**	0.24	1	>0.9999	[0.00, 0.02]
				**CourseYear**	6.34	5	0.0002	[0.10, 2.53]
				**ActivityDuration**	1.01	5	>0.9999	[0.00, 0.81]
				**METsEstimate**	1.51	1	>0.9999	[0.00, 0.11]
				**SchoolType**	175.85	1	$1.75\cdot 10^{-37}$	[3.60, 9.17]
				**CityPopulation**	114.85	1	$6.93\cdot 10^{-25}$	[1.98, 6.61]
				**CityLocation**	27.41	1	$3.93\cdot 10^{-06}$	[0.13, 2.48]
				**GLM:**	15.72	30, 2481	$5.56\cdot 10^{-72}$	[13.71, 18.74]

**Table 4:** WHOQOL: general linear model without and with control variables; statistical significance (*p* values) and effect sizes (confidence interval of Cohen’s partial eta-squared, $\eta^{2}$ in percentage) with Bonferroni correction for 5% significance level. Statistically significant differences and the presence of effect sizes are shaded in gray. Outcome is the recruitment methods (randomized or volunteers). GLM line is the overall model test (*F*, associated with $\eta^{2}=R^{2}$).

	Without control variables				With control variables			
	F	df	p	$\eta^{2}$ (%)	F	df	p	$\eta^{2}$ (%)
**WHOQOL_QoL**	0.08	1	>0.9999	[0.00, 0.00]	0.07	1	>0.9999	[0.00, 0.00]
**WHOQOL_health**	2.94	1	0.5199	[0.00, 0.74]	2.36	1	>0.9999	[0.00, 0.76]
**WHOQOL_phys**	4.86	1	0.1657	[0.00, 0.91]	0.52	1	>0.9999	[0.00, 0.04]
**WHOQOL_psy**	0.96	1	>0.9999	[0.00, 0.51]	1.56	1	>0.9999	[0.00, 0.11]
**WHOQOL_soc**	2.34	1	0.7583	[0.00, 0.68]	6.21	1	0.1914	[0.00, 1.10]
**WHOQOL_env**	0.02	1	>0.9999	[0.00, 0.00]	1.24	1	>0.9999	[0.00, 0.09]
**GLM:**	**4.51**	**6, 2544**	**0.0007**	**[0.57, 2.22]**				
				**Sex**	13.72	1	0.0032	[0.00, 1.59]
				**Age**	0.36	1	>0.9999	[0.00, 0.03]
				**BMI**	0.43	1	>0.9999	[0.00, 0.03]
				**CourseYear**	6.12	5	0.0002	[0.10, 2.39]
				**ActivityDuration**	0.96	5	>0.9999	[0.00, 0.74]
				**METsEstimate**	1.15	1	>0.9999	[0.00, 0.08]
				**SchoolType**	174.68	1	$2.04\cdot 10^{-37}$	[3.65, 8.99]
				**CityPopulation**	109.59	1	$5.97\cdot 10^{-24}$	[1.92, 6.27]
				**CityLocation**	34.57	1	$7.00\cdot 10^{-08}$	[0.26, 2.79]
				**GLM:**	19.30	23, 2488	$2.69\cdot 10^{-71}$	[12.62, 17.61]

**Table 5:** DREEM: general linear model without and with control variables; statistical significance (*p* values) and effect sizes (confidence interval of Cohen’s partial eta-squared, $\eta^{2}$ in percentage) with Bonferroni correction for 5% significance level. Statistically significant differences and the presence of effect sizes are shaded in gray. Outcome is the recruitment methods (randomized or volunteers). GLM line is the overall model test (*F*, associated with $\eta^{2}=R^{2}$).

	Without control variables				With control variables			
	F	df	p	$\eta^{2}$ (%)	F	df	p	$\eta^{2}$ (%)
**DREEM_learn**	2.92	1	0.4394	[0.00, 0.72]	1.42	1	>0.9999	[0.00, 0.10]
**DREEM_teacher**	0.91	1	>0.9999	[0.00, 0.49]	0.72	1	>0.9999	[0.00, 0.05]
**DREEM_self**	2.58	1	0.5402	[0.00, 0.68]	0.03	1	>0.9999	[0.00, 0.00]
**DREEM_env**	4.12	1	0.2124	[0.00, 0.83]	0.16	1	>0.9999	[0.00, 0.01]
**DREEM_soc**	0.27	1	>0.9999	[0.00, 0.02]	1.48	1	>0.9999	[0.00, 0.10]
**GLM:**	**3.02**	**5, 2545**	**0.0303**	**[0.25, 1.52]**				
				**Sex**	13.44	1	0.0035	[0.00, 1.59]
				**Age**	0.58	1	>0.9999	[0.00, 0.04]
				**BMI**	0.79	1	>0.9999	[0.00, 0.06]
				**CourseYear**	7.36	5	$1.02\cdot 10^{-05}$	[0.20, 2.70]
				**ActivityDuration**	1.04	5	>0.9999	[0.00, 0.77]
				**METsEstimate**	1.44	1	>0.9999	[0.00, 0.10]
				**SchoolType**	186.05	1	$9.23\cdot 10^{-40}$	[3.98, 9.41]
				**CityPopulation**	111.97	1	$1.76\cdot 10^{-24}$	[1.98, 6.34]
				**CityLocation**	26.65	1	$3.69\cdot 10^{-06}$	[0.15, 2.35]
				**GLM:**	20.41	22, 2489	$8.53\cdot 10^{-73}$	[12.72, 17.75]

**Table 6:** VERASQ: general linear model without and with control variables; statistical significance (*p* values) and effect sizes (confidence interval of Cohen’s partial eta-squared, $\eta^{2}$ in percentage) with Bonferroni correction for 5% significance level. Statistically significant differences and the presence of effect sizes are shaded in gray. Outcome is the recruitment methods (randomized or volunteers). GLM line is the overall model test (*F*, associated with $\eta^{2}=R^{2}$).

	Without control variables				With control variables			
	F	df	p	$\eta^{2}$ (%)	F	df	p	$\eta^{2}$ (%)
**VERASQ_time**	0.88	1	>0.9999	[0.00, 0.46]	0.52	1	>0.9999	[0.00, 0.04]
**VERASQ_psy**	9.62	1	0.0078	[0.01, 1.21]	0.44	1	>0.9999	[0.00, 0.03]
**VERASQ_phys**	3.57	1	0.2356	[0.00, 0.75]	0.32	1	>0.9999	[0.00, 0.02]
**VERASQ_env**	4.88	1	0.1091	[0.00, 0.86]	6.18	1	0.1689	[0.00, 1.08]
**GLM:**	**7.33**	**4, 2546**	** $5.31\cdot 10^{-05}$ **	**[0.57, 2.19]**				
				**Sex**	11.65	1	0.0085	[0.00, 1.46]
				**Age**	0.64	1	>0.9999	[0.00, 0.04]
				**BMI**	0.52	1	>0.9999	[0.00, 0.04]
				**CourseYear**	7.27	5	$1.18\cdot 10^{-05}$	[0.19, 2.66]
				**ActivityDuration**	0.99	5	>0.9999	[0.00, 0.74]
				**METsEstimate**	1.49	1	>0.9999	[0.00, 0.10]
				**SchoolType**	179.09	1	$2.23\cdot 10^{-38}$	[3.80, 9.10]
				**CityPopulation**	113.96	1	$6.25\cdot 10^{-25}$	[2.04, 6.40]
				**CityLocation**	33.38	1	$1.11\cdot 10^{-07}$	[0.25, 2.69]
				**GLM:**	21.35	21, 2490	$2.69\cdot 10^{-73}$	[12.75, 17.66]

**Table 7:** IRI: general linear model without and with control variables; statistical significance (*p* values) and effect sizes (confidence interval of Cohen’s partial eta-squared, $\eta^{2}$ in percentage) with Bonferroni correction for 5% significance level. Statistically significant differences and the presence of effect sizes are shaded in gray. Outcome is the recruitment methods (randomized or volunteers). GLM line is the overall model test (*F*, associated with $\eta^{2}=R^{2}$).

	Without control variables				With control variables			
	F	df	p	$\eta^{2}$ (%)	F	df	p	$\eta^{2}$ (%)
**IRI_empathy**	0.11	1	>0.9999	[0.00, 0.00]	2.10	1	>0.9999	[0.00, 0.71]
**IRI_perspec**	$6.8\cdot 10^{-04}$	1	>0.9999	[0.00, 0.00]	0.06	1	>0.9999	[0.00, 0.00]
**IRI_distress**	3.30	1	0.2082	[0.00, 0.69]	2.12	1	>0.9999	[0.00, 0.71]
**GLM:**	**1.29**	**3, 2547**	**0.3569**	**[0.03, 0.68]**				
				**Sex**	15.15	1	0.0012	[0.02, 1.67]
				**Age**	1.34	1	>0.9999	[0.00, 0.09]
				**BMI**	1.27	1	>0.9999	[0.00, 0.09]
				**CourseYear**	6.66	5	$4.36\cdot 10^{-05}$	[0.15, 2.49]
				**ActivityDuration**	1.00	5	>0.9999	[0.00, 0.73]
				**METsEstimate**	1.45	1	>0.9999	[0.00, 0.10]
				**SchoolType**	168.83	1	$2.55\cdot 10^{-36}$	[3.54, 8.68]
				**CityPopulation**	112.81	1	$1.01\cdot 10^{-24}$	[2.04, 6.34]
				**CityLocation**	35.60	1	$3.33\cdot 10^{-08}$	[0.30, 2.80]
				**GLM:**	21.19	20, 2491	$1.35\cdot 10^{-69}$	[12.00, 16.81]

**Table 8:** RS14: general linear model without and with control variables; statistical significance (*p* values) and effect sizes (confidence interval of Cohen’s partial eta-squared, $\eta^{2}$ in percentage) with Bonferroni correction for 5% significance level. Statistically significant differences and the presence of effect sizes are shaded in gray. Outcome is the recruitment methods (randomized or volunteers). GLM line is the overall model test (*F*, associated with $\eta^{2}=R^{2}$).

	Without control variables				With control variables			
	F	df	p	$\eta^{2}$ (%)	F	df	p	$\eta^{2}$ (%)
**RS14_selfreliance**	$7.5\cdot 10^{-03}$	1	>0.9999	[0.00, 0.00]	$5.9\cdot 10^{-04}$	1	>0.9999	[0.00, 0.00]
**RS14_meaningful**	0.01	1	>0.9999	[0.00, 0.00]	0.11	1	>0.9999	[0.00, 0.00]
**RS14_equaninity**	1.83	1	0.8804	[0.00, 0.60]	1.17	1	>0.9999	[0.00, 0.08]
**RS14_perseverance**	1.54	1	>0.9999	[0.00, 0.57]	0.17	1	>0.9999	[0.00, 0.01]
**RS14_aloneness**	0.98	1	>0.9999	[0.00, 0.50]	$3.5\cdot 10^{-04}$	1	>0.9999	[0.00, 0.00]
**GLM:**	**1.40**	**5, 2545**	**0.3082**	**[0.11, 1.02]**				
				**Sex**	14.22	1	0.0023	[0.01, 1.64]
				**Age**	0.95	1	>0.9999	[0.00, 0.07]
				**BMI**	0.99	1	>0.9999	[0.00, 0.07]
				**CourseYear**	6.34	5	0.0001	[0.12, 2.44]
				**ActivityDuration**	1.03	5	>0.9999	[0.00, 0.76]
				**METsEstimate**	1.46	1	>0.9999	[0.00, 0.10]
				**SchoolType**	169.47	1	$2.21\cdot 10^{-36}$	[3.54, 8.78]
				**CityPopulation**	110.04	1	$4.47\cdot 10^{-24}$	[1.95, 6.29]
				**CityLocation**	34.65	1	$6.26\cdot 10^{-08}$	[0.27, 2.79]
				**GLM:**	19.19	22, 2489	$4.28\cdot 10^{-68}$	[12.07, 16.85]

**Table 9:** BURNOUT: general linear model without and with control variables; statistical significance (*p* values) and effect sizes (confidence interval of Cohen’s partial eta-squared, $\eta^{2}$ in percentage) with Bonferroni correction for 5% significance level. Statistically significant differences and the presence of effect sizes are shaded in gray. Outcome is the recruitment methods (randomized or volunteers). GLM line is the overall model test (*F*, associated with $\eta^{2}=R^{2}$).

	Without control variables				With control variables			
	F	df	p	$\eta^{2}$ (%)	F	df	p	$\eta^{2}$ (%)
**BURNOUT_exhaut**	9.89	1	0.0050	[0.02, 1.19]	8.09	1	0.0538	[0.00, 1.21]
**BURNOUT_deperson**	15.99	1	0.0002	[0.10, 1.57]	1.81	1	>0.9999	[0.00, 0.68]
**BURNOUT_accomp**	6.25	1	0.0374	[0.00, 0.93]	2.58	1	>0.9999	[0.00, 0.76]
**GLM:**	**7.91**	**3, 2547**	**0.0002**	**[0.39, 1.88]**				
				**Sex**	10.64	1	0.0134	[0.00, 1.38]
				**Age**	1.11	1	>0.9999	[0.00, 0.08]
				**BMI**	1.23	1	>0.9999	[0.00, 0.09]
				**CourseYear**	6.34	5	$8.90\cdot 10^{-05}$	[0.13, 2.41]
				**ActivityDuration**	0.97	5	>0.9999	[0.00, 0.72]
				**METsEstimate**	1.54	1	>0.9999	[0.00, 0.11]
				**SchoolType**	173.67	1	$2.62\cdot 10^{-37}$	[3.68, 8.87]
				**CityPopulation**	109.58	1	$4.79\cdot 10^{-24}$	[1.95, 6.20]
				**CityLocation**	33.96	1	$7.62\cdot 10^{-08}$	[0.27, 2.71]
				**GLM:**	21.71	20, 2491	$1.99\cdot 10^{-71}$	[12.25, 17.18]

**Table 10:** PSQI: general linear model without and with control variables; statistical significance (*p* values) and effect sizes (confidence interval of Cohen’s partial eta-squared, $\eta^{2}$ in percentage) with Bonferroni correction for 5% significance level. Statistically significant differences and the presence of effect sizes are shaded in gray. Outcome is the recruitment methods (randomized or volunteers). GLM line is the overall model test (*F*, associated with $\eta^{2}=R^{2}$).

	Without control variables				With control variables			
	F	df	p	$\eta^{2}$ (%)	F	df	p	$\eta^{2}$ (%)
**PSQI_quality**	2.27	1	0.9253	[0.00, 0.69]	0.99	1	>0.9999	[0.00, 0.07]
**PSQI_latency**	0.08	1	>0.9999	[0.00, 0.00]	0.07	1	>0.9999	[0.00, 0.00]
**PSQI_duration**	0.12	1	>0.9999	[0.00, 0.00]	0.31	1	>0.9999	[0.00, 0.02]
**PSQI_efficiency**	2.25	1	0.9344	[0.00, 0.69]	1.49	1	>0.9999	[0.00, 0.11]
**PSQI_disturbance**	2.25	1	0.9351	[0.00, 0.69]	1.45	1	>0.9999	[0.00, 0.10]
**PSQI_medication**	0.02	1	>0.9999	[0.00, 0.00]	0.05	1	>0.9999	[0.00, 0.00]
**PSQI_dysfunction**	5.66	1	0.1221	[0.00, 1.00]	2.17	1	>0.9999	[0.00, 0.75]
**GLM:**	**3.40**	**7, 2543**	**0.0044**	**[0.53, 2.04]**				
				**Sex**	12.97	1	0.0052	[0.00, 1.58]
				**Age**	0.59	1	>0.9999	[0.00, 0.04]
				**BMI**	0.87	1	>0.9999	[0.00, 0.06]
				**CourseYear**	6.06	5	0.0002	[0.09, 2.40]
				**ActivityDuration**	0.96	5	>0.9999	[0.00, 0.75]
				**METsEstimate**	1.57	1	>0.9999	[0.00, 0.11]
				**SchoolType**	164.61	1	$2.50\cdot 10^{-35}$	[3.38, 8.63]
				**CityPopulation**	112.07	1	$1.92\cdot 10^{-24}$	[1.98, 6.42]
				**CityLocation**	35.41	1	$4.87\cdot 10^{-08}$	[0.28, 2.86]
				**GLM:**	18.02	24, 2487	$1.08\cdot 10^{-68}$	[12.38, 17.27]

The variables of interest in Table 3 are the global scores of the questionnaires. There is no effect of recruitment (randomized or volunteer groups), except for Maslach Burnout Inventory (Table 3, left panel). However, with the addition of control variables the difference between randomized and volunteers responses are mainly affected by the type of school (public or private), the size of the city population, and the school’s location (rural or metropolitan). These variables showed the largest effect sizes and are attributable to differences in questionnaire response patterns (in this example, only Burnout). Table 1 reveals that the convenience sample had a higher proportion of volunteers from private schools (presumably fee-paying students with better financial conditions), living in larger cities and in schools located in rural regions (Table 11 shows what differences are statistically significant). This example illustrates that, if statistical analysis were performed without accounting for variables known to influence questionnaire responses, we would risk identifying associations that could misleadingly suggest causal explanations for the observed differences. This does not deny that there is an average difference in the response pattern to the Burnout questionnaire or that the questionnaire is inadequate in its measurement (Table 2), but rather emphasizes that the type of school and the city of residence may play a dominant role in affecting the quality of life of students (in this case, specifically influencing the Burnout scores).

**Table 11:** Estimated marginal means for main control variables: emmean indicates the adjusted proportion of volunteers and randomized respondents; cld shows the compact letter display of all pairwise comparisons to indicate grouping similarity; significant differences are reported with Bonferroni correction among categories.

	emmean:	Volunteer	Randomized	cld	$\eta^{2}$ (%)
**Sex**	Female	53.8%	46.2%	a	[0.09,1.72]
	Male	45.7%	54.3%	b	
**Course year**	1	51.9%	48.1%	a	[0.27,2.46]
	2	56.0%	44.0%	a	
	3	56.1%	43.9%	a	
	4	49.3%	50.7%	a	
	5	46.7%	53.3%	ab	
	6	38.8%	61.2%	b	
**School type**	Public	36.5%	63.5%	a	[3.74,8.3]
	Private	63.1%	36.9%	b	
**City population**	< 500 thousands	43.1%	56.9%	a	[2.58,6.63]
	500 th. - 1 million	38.5%	61.5%	a	
	1 million - 5 millions	44.6%	55.4%	a	
	> 5 millions	72.9%	27.1%	b	
**City location**	Capital	44.1%	55.9%	a	[0.02,1.38]
	Non-capital	55.5%	44.5%	b	

For this particular example, Table 9 details Burnout questionnaire, showing their domains as variables of interest. Statistical differences were observed in all three domains of Emotional Exhaustion (higher among volunteers), Depersonalization (higher among randomized students), and Reduced Personal Accomplishment domain (higher among randomized students) as shown in Table 2. However the Reduced Personal Accomplishment domain has negligible effect size (it includes zero in the confidence interval).

Similarly, the psychological subdomain of VERAS-Q (Table 6) showed effect of recruitment method, which vanished when control variables were included. In all other questionnaires no mean difference was observed between randomized and volunteer respondents (Tables 4, 5, 7, 8, and 10). Nevertheless, the control variables revealed impact on the response patterns of randomized versus volunteer students, usually in the following order of importance (i.e., effect size): type of school, city population, city location, medical school year, and sex (with negligible effect size) for WHOQOL, DREEM, IRI, RS14, and PSQI.

Therefore, it is suggested that the difference between the two groups lies in these five control variables. The recruitment effect, if it exists, is not detectable by the questionnaires. Thus, it is necessary to check if the randomized and volunteer student groups differ in one or more of the control variables using a *post hoc* test with estimated marginal means (R packages emmeans [51] and multcomp [52]). Table 11 shows that the groups differ in all these variables, but not in all categories: there are more women among volunteers and more men among randomized participants. A trend was observed for volunteers to be more frequent in the early years of medical school, gradually shifting so that responses from the final years required active recruitment by randomization. In the three categories of smaller city populations, randomized participants were more prevalent, whereas in cities over 5 million inhabitants, volunteers predominated. Finally, more randomized participants were from schools located in capitals while more volunteers came from schools located in rural areas.

## Discussion

This study constitutes a groundbreaking initiative in Brazil and could be one of the first globally to utilize a distinctive sampling technique, innovative data collection strategies, and widespread participation from numerous schools and students. The instruments used in the present study have been previously described in publications from our research group with details on validation, scoring, and properties; namely, WHOQOL, Burnout, and IRI [29]; DREEM, WHOQOL, BDI, and RS-14 [30]; DREEM, WHOQOL, GCol, and CQol [31]; BDI and STAI [32]; WHOQOL, VERASQ, and METs [53]; and PSQI and Epworth [33]. In these studies the results of the randomly selected medical students have been previously published, but the data from volunteers have not. Here, there was a rare opportunity for comparison between recruitment strategies, having a data collection platform that obtained two large samples of comparable sizes of a convenience sample while simultaneously encouraging randomized students to respond to the questionnaires. Consequently, this enabled a more robust and meaningful comparison between the two groups. Given that the opportunity to collect the analyzed data preceded the substantial increase in medical schools in the country, this study can serve both as a baseline for comparison with future measurements and as a source of recommendations for the most effective strategies designed to reach a sufficient number of students.

SWOT analysis showed, as expected, that the large size of the Brazilian country and the unreliability of the telecommunication infrastructure hindered the study somewhat, as well as the small core research team, local researchers’ work overload and difficulties in motivating students to answer a large number of items. Perhaps, our relatively small losses may be related to our main strengths, which were threefold: support network (provided remotely by the core group to local researchers, and by personal contact between local researchers and volunteers), institutional support, and a customized electronic platform for data collection and follow-up. These strategies were a mixture of the electronic platform customization and successful human-driven actions.

Somewhat surprisingly, the number of volunteers was close to that of randomized respondents. In this study, we lack sufficient information to understand why this was the case. Concretely, many volunteers that started answering completed the all set of questions. On the other hand, we can only speculate on their motivations. Perhaps, volunteers had additional factors propelling them to undertake the task; it is conceivable that the intensive campaign was perceived by volunteers who, trusting the researchers, chose to contribute; or the electronic platform may have been considered attractive as it provided feedback at the conclusion of all responses, thus encouraging adherence.

A risk for the current study was the substantial volume of questions. While it may be more convenient to collect and analyze data through electronic systems, there is a risk of losing sight of the overall number of questions that respondents need to answer. As noted by Smith and Huntington [54], studies with 80 to 200 items experience a 10 to 15% loss due to respondent exhaustion, and those with over 200 items have a significantly reduced response rate. Therefore, all the already mentioned efforts to reach randomized students may be what contributed to achieve 81.8% of adherence (1350 out of 1650 respondents), a remarkably high proportion.

The question, therefore, is whether such effort pays off. Our expectation, in advocating for randomization, was to secure more reliable data with fewer biases concerning the perceptions of medical students. It is often assumed that students with poorer quality of life perceptions are more likely to volunteer, yet our findings do not support this. Randomization efforts are often associated with smaller samples and number of instruments, while larger samples and extensive evaluations tend to rely on convenience. For example, Mohammadi et al. [55] randomized 60 paramedics to assess the impact of bioethical education using two questionnaires (adherence or completion rates are not reported, which limits the evaluation of participant engagement). In contrast, a survey including 3,330 respondents out of 10,622 invitations (31% adherence), which applied multiple instruments to assess functional status, mental health, health literacy, social support, among other domains, used convenience sampling [56].

In general, studies rely on convenience samples. Quality of life among students and health professionals continues to appear in more recent publications, indicating that this concern is not a resolved issue. Examples include the application of WHOQOL-BREF showing that, among health students (n=349), dentistry students tend to report higher quality of life compared to pharmacy and nursing students, younger students in the early years showing lower scores [37]; and among nursing students (n=147) reporting declines in some domains using descriptive statistics and Pearson’s correlation [38]. Quality of life was considered moderate among health professionals (n=1850) in the Gaza Strip [39]. Using various instruments, nursing students (n=341) showed burnout and traumatic stress related to the type of environment in which activities are carried out [40]. Among medical students evaluated before and after the COVID-19 pandemic in Austria, increased symptoms of depressive and anxiety were observed, together with a decrease in subjective well-being and the perception of increased study load and stress. The study also indicated that the study year is a predictor of quality of life, with the early years associated with worse indicators. In our study, the group is homogeneous in terms of environments, since all medical students are exposed to a variety of settings, including outpatient care, wards, and emergency departments, throughout their training, with the emphasis shifting progressively from outpatient care to emergency attendance as they approach the end of the course. However, in our study, we did not find a clear and statistically significant trend related to the isolated effect of the study year on the questionnaire results among randomized or volunteer students, despite having considerable sample sizes.

Interestingly, we did not find important differences between the measurements obtained from the questionnaires, either in their overall scores or in their specific subdomains, when comparing the groups of randomized students and those who voluntarily responded to the questionnaires. What we identified were differences in the composition of the groups, leading to a higher proportion of women, fewer students in the later years of the course, more students from private schools, and more students from outside the capitals among the volunteers.

This study comes with certain limitations that need to be acknowledged before any interpretation. For instance, when observing an increase in exhaustion or a decrease in the perception of environmental quality with advancing course years, especially if it aligns with our preconceptions regarding the escalating stress in medical education, there might be a temptation to infer a causal explanation. Although this study stands among the largest conducted to date, we must emphasize that it adopts a cross-sectional approach. While this awareness is not new in the realm of science, it is crucial to recognize that it constitutes a potential source of cognitive biases. Additionally, from a statistical standpoint, the size of the respondent pool can easily yield significant differences in p-values, as they are highly sensitive to larger sample sizes. Consequently, even when p-values indicate statistical significance, it is imperative to critically evaluate the practical significance of each finding. Conversely, despite the substantial number of respondents, no significant differences were observed concerning the questionnaire measurements with respect to the recruitment methods.

The ultimate choice between a randomized or convenience sample hinges on one’s objectives. Even if potential bias exists, opting for convenience sampling based on volunteers, considering the worst-case scenario for a situational diagnosis, is justified. However, biases are more likely to occur in smaller samples. In larger samples, such as the one in the present study, personal bias may be diluted, resulting in similarity between the two student groups. Smaller studies may benefit from a bias toward overestimating a negative scenario, especially if it leads to interventions aimed at improving the quality of life, fostering a healthier environment, or enhancing relationships that could benefit all students, including those who may not respond to questions due to a more optimistic outlook. However, determining the effectiveness of any intervention may become challenging. It is well-established that individuals under burnout or depression, even after recovery, may persist with more negative views or express more critical opinions for an extended period, potentially longer than the duration of the medical course. Consequently, measuring outcomes among volunteers may erroneously show no effect of an intervention, leading to the abandonment of promising initiatives.

The present analysis includes only a limited number of control variables intended to address the observed differences between randomized and voluntary participants, thereby lessening the potential impact that the questionnaires might have revealed. One of the purposes of randomization is to eliminate the need for controls, assuming that perturbations by confounding variables are randomly dispersed and, consequently, their effects are neutralized in the final results. This rationale underlies the previous publications of our group, which focused solely on the 1350 randomized subjects. Opting for convenience samples necessitates the collection of additional data as potential controls, along with corresponding statistical analyses. Our findings suggest that, in large samples, life circumstances influence participation but do not significantly affect questionnaire outcomes.

Since the present study suggests that the two recruitment methods did not lead to disparate conclusions, studies using samples obtained through probabilistic and nonprobabilistic methods may be integrated in the future, and initial proposals in this regard already exist in the literature [57]. Sustainability of the research network is required to ensure future longitudinal studies and deepen our understanding of the universe of medical students, in order to contribute to the development and improvement of the medical school curricula.

## Conclusion

These are the ten lessons learned from the VERAS project which can serve as guidelines to researchers interested in designing similar studies:

1. A user-friendly online platform enabled recruitment at reduced cost;
2. Random presentation of questionnaires is assumed to mitigate order effects;
3. Respondents must be able to pause and resume lengthy questionnaires;
4. Selection of questionnaires is essential to meet study objectives;
5. Immediate personal feedback is ethically advisable;
6. The electronic system enforced sequential completion, preventing missing data and incomplete responses;
7. Local researchers were required to increase adherence of respondents;
8. Weekly progress reports may motivate local researchers and support network cohesion;
9. Respondents should be informed and able to consult local researchers for support;
10. The combination of local researchers’ engagement and web-based accessibility likely contributed to large sample sizes.

Finally, our data suggest that the use of convenience samples may not introduce biases to study results. Therefore, the decision to randomize or persuade students to answer questionnaires should be left to the discretion of each researcher.

## References

[1] Dyrbye LN, Thomas MR, Huntington JL, Lawson KL, Novotny PJ, Sloan JA, et al. Personal life events and medical student burnout: a multicenter study. Acad Med. 2006;81(4):374–84. doi: 10.1097/00001888-200604000-00010 16565189

[2] Roh M-S, Jeon HJ, Kim H, Cho HJ, Han SK, Hahm B-J. Factors influencing treatment for depression among medical students: a nationwide sample in South Korea. Med Educ. 2009;43(2):133–9. doi: 10.1111/j.1365-2923.2008.03255.x 19161483

[3] Dyrbye LN, Harper W, Durning SJ, Moutier C, Thomas MR, Massie FS Jr, et al. Patterns of distress in US medical students. Med Teach. 2011;33(10):834–9. doi: 10.3109/0142159X.2010.531158 21942482

[4] Thomas MR, Dyrbye LN, Huntington JL, Lawson KL, Novotny PJ, Sloan JA, et al. How do distress and well-being relate to medical student empathy? A multicenter study. J Gen Intern Med. 2007;22(2):177–83. doi: 10.1007/s11606-006-0039-6 17356983 PMC1824738

[5] Ross KN. Sample design for educational survey research: Module 3. UNESCO Digital Library; 2005. https://unesdoc.unesco.org/ark:/48223/pf0000214550

[6] Tashakkori A, Teddlie C. SAGE Handbook of Mixed Methods in Social & Behavioral Research. SAGE Publications, Inc.; 2010. 10.4135/9781506335193

[7] Dyrbye LN, Thomas MR, Huschka MM, Lawson KL, Novotny PJ, Sloan JA, et al. A multicenter study of burnout, depression, and quality of life in minority and nonminority US medical students. Mayo Clin Proc. 2006;81(11):1435–42. doi: 10.4065/81.11.1435 17120398

[8] Dyrbye LN, Thomas MR, Massie FS, Power DV, Eacker A, Harper W, et al. Burnout and suicidal ideation among U.S. medical students. Ann Intern Med. 2008;149(5):334–41. doi: 10.7326/0003-4819-149-5-200809020-00008 18765703

[9] Dyrbye LN, Thomas MR, Harper W, Massie FS Jr, Power DV, Eacker A, et al. The learning environment and medical student burnout: a multicentre study. Med Educ. 2009;43(3):274–82. doi: 10.1111/j.1365-2923.2008.03282.x 19250355

[10] Dyrbye LN, Thomas MR, Power DV, Durning S, Moutier C, Massie FS Jr, et al. Burnout and serious thoughts of dropping out of medical school: a multi-institutional study. Acad Med. 2010;85(1):94–102. doi: 10.1097/ACM.0b013e3181c46aad 20042833

[11] Dyrbye LN, Massie FS Jr, Eacker A, Harper W, Power D, Durning SJ, et al. Relationship between burnout and professional conduct and attitudes among US medical students. JAMA. 2010;304(11):1173–80. doi: 10.1001/jama.2010.1318 20841530

[12] Dyrbye LN, Harper W, Moutier C, Durning SJ, Power DV, Massie FS, et al. A multi-institutional study exploring the impact of positive mental health on medical students’ professionalism in an era of high burnout. Acad Med. 2012;87(8):1024–31. doi: 10.1097/ACM.0b013e31825cfa35 22722352

[13] Boor K, Scheele F, van der Vleuten CPM, Teunissen PW, den Breejen EME, Scherpbier AJJA. How undergraduate clinical learning climates differ: a multi-method case study. Med Educ. 2008;42(10):1029–36. doi: 10.1111/j.1365-2923.2008.03149.x 18823522

[14] van Hell EA, Kuks JBM, Cohen-Schotanus J. Time spent on clerkship activities by students in relation to their perceptions of learning environment quality. Med Educ. 2009;43(7):674–9. doi: 10.1111/j.1365-2923.2009.03393.x 19573191

[15] Hillis JM, Perry WRG, Carroll EY, Hibble BA, Davies MJ, Yousef J. Painting the picture: Australasian medical student views on wellbeing teaching and support services. Med J Aust. 2010;192(4):188–90. doi: 10.5694/j.1326-5377.2010.tb03476.x 20170454

[16] Warnecke E, Quinn S, Ogden K, Towle N, Nelson MR. A randomised controlled trial of the effects of mindfulness practice on medical student stress levels. Med Educ. 2011;45(4):381–8. doi: 10.1111/j.1365-2923.2010.03877.x 21401686

[17] Roh M-S, Hahm B-J, Lee DH, Suh DH. Evaluation of empathy among Korean medical students: a cross-sectional study using the Korean Version of the Jefferson Scale of Physician Empathy. Teach Learn Med. 2010;22(3):167–71. doi: 10.1080/10401334.2010.488191 20563934

[18] Goebert D, Thompson D, Takeshita J, Beach C, Bryson P, Ephgrave K, et al. Depressive symptoms in medical students and residents: a multischool study. Acad Med. 2009;84(2):236–41. doi: 10.1097/ACM.0b013e31819391bb 19174678

[19] Reed DA, Shanafelt TD, Satele DW, Power DV, Eacker A, Harper W, et al. Relationship of pass/fail grading and curriculum structure with well-being among preclinical medical students: a multi-institutional study. Acad Med. 2011;86(11):1367–73. doi: 10.1097/ACM.0b013e3182305d81 21952063

[20] Guimarães RA, de França E Silva ALG, de Souza MR, Guimarães AM, de Souza Lauro ME, Naghettini AV, et al. Trend and spatial clustering of medical education in Brazil: an ecological study of time series from 2010 to 2021. BMC Health Serv Res. 2023;23(1):882. doi: 10.1186/s12913-023-09795-9 37608336 PMC10464021

[21] Tempski P, Bellodi PL, Paro HBMS, Enns SC, Martins MA, Schraiber LB. What do medical students think about their quality of life? A qualitative study. BMC Med Educ. 2012;12:106. doi: 10.1186/1472-6920-12-106 23126332 PMC3527341

[22] Nassif ACN. Escolas MÂ´edicas do Brasil: portal de cursos de medicina no Brasil. [cited 2025 August 23]. https://www.escolasmedicas.com.br/

[23] Duvivier RJ, Boulet JR, Opalek A, van Zanten M, Norcini J. Overview of the world’s medical schools: an update. Med Educ. 2014;48(9):860–9. doi: 10.1111/medu.12499 25113113

[24] Instituto Nacional de Estudos e Pesquisas Educacionais Anísio Teixeira (Inep). INEP/Ministério da Educação. 2025. https://www.gov.br/inep/pt-br/acesso-a-informacao/dados-abertos/sinopses-estatisticas/educacao-superior

[25] Wolff A. Demografia médica 2025 : como está o cenário da graduação em medicina no Brasil. Estratégia MED. 2025. [cited 2025 August 26]. https://med.estrategia.com/portal/atualidades/demografia-medica-2025-como-esta-o-cenario-da-graduacao-em-medicina-no-brasil/

[26] Avena KM, Quintanilha LF, Luzardo Filho RL, Andrade BB. Lessons learned from the expansion of medical schools in Brazil: a review of challenges and opportunities. Front Educ. 2025;9. doi: 10.3389/feduc.2024.1494445

[27] Andrade BB. The dark side of private medical education in Brazil. Front Med (Lausanne). 2025;12:1504794. doi: 10.3389/fmed.2025.1504794 40034387 PMC11873559

[28] Tempski P, Girotto LC, Brenelli S, Giamberardino DD, Martins MA. Accreditation of medical education in Brazil: an evaluation of seventy-six medical schools. BMC Med Educ. 2024;24(1):656. doi: 10.1186/s12909-024-05623-8 38867222 PMC11167757

[29] Paro HBMS, Silveira PSP, Perotta B, Gannam S, Enns SC, Giaxa RRB, et al. Empathy among medical students: is there a relation with quality of life and burnout?. PLoS One. 2014;9(4):e94133. doi: 10.1371/journal.pone.0094133 24705887 PMC3976378

[30] Tempski P, Santos IS, Mayer FB, Enns SC, Perotta B, Paro HBMS, et al. Relationship among Medical Student Resilience, Educational Environment and Quality of Life. PLoS One. 2015;10(6):e0131535. doi: 10.1371/journal.pone.0131535 26121357 PMC4486187

[31] Enns SC, Perotta B, Paro HB, Gannam S, Peleias M, Mayer FB, et al. Medical students’ perception of their educational environment and quality of life: is there a positive association?. Acad Med. 2016;91(3):409–17. doi: 10.1097/ACM.0000000000000952 26556293

[32] Brenneisen Mayer F, Souza Santos I, Silveira PSP, Itaqui Lopes MH, de Souza ARND, Campos EP, et al. Factors associated to depression and anxiety in medical students: a multicenter study. BMC Med Educ. 2016;16(1):282. doi: 10.1186/s12909-016-0791-1 27784316 PMC5080800

[33] Perotta B, Arantes-Costa FM, Enns SC, Figueiro-Filho EA, Paro H, Santos IS, et al. Sleepiness, sleep deprivation, quality of life, mental symptoms and perception of academic environment in medical students. BMC Med Educ. 2021;21(1):111. doi: 10.1186/s12909-021-02544-8 33596885 PMC7890911

[34] Castaldelli-Maia JM, Lewis T, Marques Dos Santos N, Picon F, Kadhum M, Farrell SM, et al. Stressors, psychological distress, and mental health problems amongst Brazilian medical students. Int Rev Psychiatry. 2019;31(7–8):603–7. doi: 10.1080/09540261.2019.1669335 31612743

[35] Silveira de Resende M, Santos IM, de Moura EC, de Almeida Pedro R, Gobbo Jr M. Impact of medical school on quality of life and mental health in Brazil: a cross-sectional comparative study. BMJ Open. 2025;15(6):e097917. doi: 10.1136/bmjopen-2024-097917 40467311 PMC12142097

[36] Moutinho ILD, Lucchetti ALG, Ezequiel O da S, Lucchetti G. Mental health and quality of life of Brazilian medical students: Incidence, prevalence, and associated factors within two years of follow-up. Psychiatry Res. 2019;274:306–12. doi: 10.1016/j.psychres.2019.02.041 30831455

[37] Galgam G, et al. Quality of life among nursing students in selected African countries. BMC Nursing. 2024;23:123. doi: 10.1186/s12912-024-01662-138360601

[38] Putri GP, Inggrini I, Tanjung NAD, Pakpahan M, Purimahua DI. The lifestyle and quality of life among nursing students. J Holist Nurs. 2025;43(1):18–25. doi: 10.1177/08980101241292208 39474641

[39] Younis J, Wang L, Abed A, Jiang H, Fan Y, Li Z, et al. Quality of life among healthcare workers in the hospitals and primary healthcare centers in Gaza Strip: a cross-sectional study. BMC Psychol. 2025;13(1):69. doi: 10.1186/s40359-025-02386-9 39856745 PMC11763158

[40] Chachula KM. Professional quality of life factors and relationships in nursing and psychiatric nursing students: an exploratory study. SAGE Open Nurs. 2021;7:2377960821994394. doi: 10.1177/2377960821994394 33912669 PMC8047937

[41] Huber A, Rabl L, Höge-Raisig T, Höfer S. Well-being, mental health, and study characteristics of medical students before and during the pandemic. Behav Sci (Basel). 2023;14(1):7. doi: 10.3390/bs14010007 38275349 PMC10812729

[42] Pacheco JP, Giacomin HT, Tam WW, Ribeiro TB, Arab C, Bezerra IM, et al. Mental health problems among medical students in Brazil: a systematic review and meta-analysis. Braz J Psychiatry. 2017;39(4):369–78. doi: 10.1590/1516-4446-2017-2223 28876408 PMC7111407

[43] Soares SJB, Fernandes CFG, Tabalipa R, Kogima F, Jubini MAM, Dias IMV, et al. Common mental disorders among medical students: systematic review and meta-analysis of Brazilian studies. Sao Paulo Med J. 2022;140(4):615–22. doi: 10.1590/1516-3180.2021.0851.R1.27012022 35946680 PMC9491479

[44] Solis AC, Lotufo-Neto F. Predictors of quality of life in Brazilian medical students: a systematic review and meta-analysis. Braz J Psychiatry. 2019;41(6):556–67. doi: 10.1590/1516-4446-2018-0116 30994854 PMC6899364

[45] Martins CK, Cruz JC, Dellalibera-Joviliano R. Quality of life in Brazilian medical students: a systematic review and meta-analysis. Trends Psychiatry Psychother. 2024;46:e20220497. doi: 10.47626/2237-6089-2022-0497 35944099 PMC11140770

[46] Erschens R, Keifenheim KE, Herrmann-Werner A, Loda T, Schwille-Kiuntke J, Bugaj TJ, et al. Professional burnout among medical students: systematic literature review and meta-analysis. Med Teach. 2019;41(2):172–83. doi: 10.1080/0142159X.2018.1457213 29656675

[47] Winter ML, Olivia SG. A scoping review of mental health needs and challenges among medical students within South African Universities. Int J Environ Res Public Health. 2024;21(5):593. doi: 10.3390/ijerph21050593 38791806 PMC11120686

[48] Cochran WG. Double sampling. 3rd ed. New York: John Wiley & Sons, Inc.; 1977.

[49] Zygmont CS. Managing the assumption of normality within the general linear model with small samples: guidelines for researchers regarding if, when and how. TQMP. 2023;19(4):302–32. doi: 10.20982/tqmp.19.4.p302

[50] Casebeer A. Application of SWOT analysis. Br J Hosp Med. 1993;49(6):430–1. 8472105

[51] Lenth RV. Emmeans: estimated marginal means, aka least-squares means. CRAN package documentation. 2023. https://CRAN.R-project.org/package=emmeans

[52] Hothorn T, Bretz F, Westfall P. Simultaneous inference in general parametric models. Biom J. 2008;50(3):346–63. doi: 10.1002/bimj.200810425 18481363

[53] Peleias M, Tempski P, Paro HB, Perotta B, Mayer FB, Enns SC, et al. Leisure time physical activity and quality of life in medical students: results from a multicentre study. BMJ Open Sport Exerc Med. 2017;3(1):e000213. doi: 10.1136/bmjsem-2016-000213 28761706 PMC5530174

[54] Smith DJ, Huntington J. Choosing the “correct” assessment tool. Curr Probl Cancer. 2006;30(6):272–82. doi: 10.1016/j.currproblcancer.2006.08.005 17123879

[55] Mohammadi MMD, Sheikhasadi H, Mahani SA, Taheri A, Sheikhbardsiri H, Abdi K. The effect of bio ethical principles education on ethical attitude of prehospital paramedic personnel. J Educ Health Promot. 2021;10:289. doi: 10.4103/jehp.jehp_708_20 34667789 PMC8459844

[56] Chamberlain AM, Hade EM, Haller IV, Horne BD, Benziger CP, Lampert BC, et al. A large, multi-center survey assessing health, social support, literacy, and self-management resources in patients with heart failure. BMC Public Health. 2024;24(1):1141. doi: 10.1186/s12889-024-18533-7 38658888 PMC11040866

[57] Wiśniowski A, Sakshaug JW, Perez Ruiz DA, Blom AG. Integrating probability and nonprobability samples for survey inference. Journal of Survey Statistics and Methodology. 2020;8(1):120–47. doi: 10.1093/jssam/smz051

[58] Natural Earth. Free vector and raster map data. 2020. [cited 2025 August 20]. https://www.naturalearthdata.com/

